# Lipase and factor V (but not viral load) are prognostic factors for the evolution of severe yellow fever cases

**DOI:** 10.1590/0074-02760190033

**Published:** 2019-05-20

**Authors:** Luciana Vilas Boas Casadio, Ana Paula Moreira Salles, Fernanda de Mello Malta, Gabriel Fialkovitz Leite, Yeh-Li Ho, Michele Soares Gomes-Gouvêa, Luiz Marcelo Sá Malbouisson, Anna S Levin, Raymundo Soares de Azevedo, Flair José Carrilho, Ana Catharina Seixas Santos Nastri, João Renato Rebello Pinho

**Affiliations:** 1 Universidade de São Paulo, Faculdade de Medicina da São Paulo, Instituto de Medicina Tropical, Departamento de Gastroenterologia, Laboratório de Gastroenterologia e Hepatologia Tropical - LIM/07, São Paulo, SP, Brasil; 2 Faculdade de Medicina da Universidade de São Paulo, Hospital das Clínicas, Departamento de Moléstias Infecciosas e Parasitárias, São Paulo, SP, Brasil; 3 Faculdade de Medicina da Universidade de São Paulo, Hospital das Clínicas, Departamento de Gastroenterologia, São Paulo, SP, Brasil; 4 Faculdade de Medicina da Universidade de São Paulo, Departamento de Patologia, São Paulo, SP, Brasil; 5 Hospital Israelita Albert Einstein, Albert Einstein Medicina Diagnóstica, Laboratório de Técnicas Especiais, São Paulo, SP, Brasil

**Keywords:** yellow fever, lipase, factor v, viral load

## Abstract

**BACKGROUND:**

Despite a highly efficacious vaccine, yellow fever (YF) is still a major threat in developing countries and a cause of outbreaks. In 2018, the Brazilian state of São Paulo witnessed a new YF outbreak in areas where the virus has not been detected before.

**OBJECTIVE:**

The aim is to describe the clinical and laboratorial characteristics of severe cases of YF, evaluate viral to determine markers associated with fatal outcome.

**METHODS:**

Acute severe YF cases (n = 62) were admitted to the Intensive Care Unit of a reference hospital and submitted to routine laboratorial evaluation on admission. YFV-RNA was detected in serum and urine by reverse transcription-quantitative polymerase chain reaction (RT-qPCR) and then sequenced. Patients were classified in two groups: survival or death.

**FINDINGS:**

In the univariate analysis the following variables were associated with outcome: alanin aminotransferase (ALT), aspartat aminotransferase (AST), AST/ALT ratio, total bilirubin (TB), chronic kidney disease epidemiology collaboration (CKD-EPI), ammonia, lipase, factor V, international normalised ratio (INR), lactate and bicarbonate. Logistic regression model showed two independent variables associated with death: lipase [odds ratio (OR) 1.018, 95% confidence interval (CI) 1.007 to 1.030, p = 0.002], and factor V (OR -0.955, 95% CI 0.929 to 0.982, p = 0.001). The estimated lipase and factor V cut-off values that maximised sensitivity and specificity for death prediction were 147.5 U/L [area under the curve (AUC) = 0.879], and 56.5% (AUC = 0.913).

**MAIN CONCLUSIONS:**

YF acute severe cases show a generalised involvement of different organs (liver, spleen, heart, kidneys, intestines and pancreas), and different parameters were related to outcome. Factor V and lipase are independent variables associated with death, reinforcing the importance of hemorrhagic events due to fulminant liver failure and pointing to pancreatitis as a relevant event in the outcome of the disease.

Yellow fever (YF) is a viral haemorrhagic fever endemic in tropical regions of Africa and Americas that affects mainly humans and nonhuman primates (NHP).^1^ Its etiological agent, the yellow fever virus (YFV), is classified in the genus *Flavivirus*, family Flaviviridae and is transmitted by infected mosquitoes. YF outbreaks cause epidemics of a potentially fatal disease due to severe haemorrhagic phenomena.

Although a highly efficacious vaccine against YFV has been available for almost a century, YF outbreaks still occur, especially in developing countries with large equatorial or tropical rainforests. These countries sometimes have low vaccine coverage due to economic reasons and to intrinsic difficulties to access the population that lives in sylvatic areas.^2^ After an incubation period of three to six days, the disease progresses in three different stages: infection (with viraemia, fever, headache and other symptoms), remission (duration up to two days with disappearance of symptoms), and intoxication (that occurs in only 15% to 20% of patients, starting between days 3 and 6 after the onset of symptoms and leads to haemorrhagic fever and multiple organ dysfunction).^1^ ^,^ ^3^ ^,^ ^4^ ^,^ ^5^ Studies with NHP detected viscerotropic activity affecting not only the liver but also other organs such as heart, kidneys, thymus, and spleen. In rodents, encephalitis also has been described. The diagnosis is classically based on serology with the detection of IgM antibodies characterising the acute phase of the disease. It is classically described that antibodies can be detected in the intoxication phase, but viraemia was not generally detected when classical viral cultivation methods were used.^1^ ^,^ ^4^ ^,^ ^6^ These data are based on ancient cohorts in times and places in which medical resources were scarce and precarious.^7^ ^,^ ^8^

At the beginning of the 20th century, massive vector control ensured the almost entire eradication of YFV, except in Africa and some few cases in the north of South America.^9^ The YF virus, the mosquito vectors, and the natural hosts, NHP, cohabit in the jungle areas. Sometimes, human cases are described in areas adjacent to jungles. These cases can also serve as a source for new infections and major outbreaks if there are susceptible individuals in the region.^10^

Such events have been recently described throughout the world. In Africa, different YF outbreaks have been described during this century. An YF outbreak in Angola began in December 2015 in its capital Luanda and there were 4,306 suspected cases and 376 deaths with a mortality rate of 8.8%.^11^ Together, Angola and the Democratic Republic of Congo, until October 28, 2016 had 962 cases of YF with 393 deaths.^12^

In Brazil, YF outbreaks have been described in the South and Southeast regions, outside the previously recognized endemic areas for YF (Amazon Region and surroundings areas). Previously, the state of São Paulo was not considered an area of risk for YF transmission, with a few cases reported occasionally, such as the outbreak in 2009 with 11 deaths.^13^ The most recent Brazilian outbreak started in 2016. Until October 2018, 1,376 confirmed YF cases and 483 deaths. From these, 555 confirmed YF cases and 203 deaths only in the state of São Paulo.^13^ ^,^ ^14^ ^,^ ^15^ ^,^ ^16^

In this epidemic of YF clinical and virological aspects could be studied using clinical means and laboratorial assays that have not been described in previous outbreaks. In our study, we included all patients who were admitted to Intensive Care Units (ICUs) of Hospital das Clínicas, University of São Paulo Medical School. during the 2018 YF outbreak. The aim was to describe the clinical and laboratorial characteristics of severe cases of YF, evaluate viral parameters such as viral load and genotype among these cases, and determine markers associated with fatal outcome. We emphasize that the laboratory methods used in this study were not available in other previous outbreaks in our region.

## SUBJECTS AND METHODS

*Patients* - Ninety-four patients with suspected YF who met the severity criteria established in our service were admitted to ICUs. Severity criteria were defined by a consensus meeting involving specialists from different clinical and surgical areas in our hospital designated to organise the emergency care of these patients. Such criteria did not exist before because the hospital had never experienced a similar situation before. The severity criteria were: alanine aminotransferase (ALT) or aspartate aminotransferase (AST) > 3,000 U/L; and/or international normalised ratio (INR) > 1.5; and/or platelets < 90,000/mm^3^; and/or renal dysfunction; and/or haemorrhagic phenomena; and/or encephalopathy; and/or clinical instability. Clinical parameters were retrospectively collected from the patients’ medical records.

*Laboratorial assays* - Blood and urine samples were collected from all patients. General clinical laboratory assays described below were carried out for diagnosis and to monitor patients’ follow up. Hepatitis A, B and C viral infections were ruled out by routine serological and molecular assays. HIV infection was also ruled out in all patients using similar assays. YFV-RNA was assayed in serum and urine samples by reverse transcription-quantitative polymerase chain reaction (RT-qPCR).

The following laboratorial assays were carried out on admission: creatinine, ammonia, lipase, total bilirubin (TB), lactate, bicarbonate, AST, ALT, INR, and factor V. These assays were carried out by routine procedures at the Clinical Laboratory Division at Hospital das Clínicas.

Chronic kidney disease epidemiology collaboration (CKD-EPI) equation was utilised for estimating glomerular filtration rate (GFR) based on serum creatinine levels, age, race, and sex, as previously described.^17^ AST/ALT ratios were also calculated.

*Detection and quantification of YFV-RNA in serum and urine samples* - YFV-RNA was isolated from 250 μL of serum and urine samples using TRIzol LS Reagent following the manufacturer’s instructions (Invitrogen, Thermo Fisher Scientific Brand, Carlsbad, CA, USA), and 20 μg of RNAse free glycogen was added to the aqueous phase as an RNA carrier.

YFV RT-qPCR was carried out using SuperScript III One-Step RT-qPCR System with Platinum Taq DNA Polymerase (Invitrogen, Thermo Fisher Scientific Brand, Carlsbad, CA, USA). The YFV 5 ´ non-coding region was targeted using specific primers and probe, as previously described.^18^

A synthetic DNA standard curve was used to quantify YFV genomes. Each assay was designed in order to include negative and positive controls, the standard curve, and reference controls. For generating the synthetic standard curves, a 95 bp oligonucleotide from YFV 5’NCR was customized (5’- CTGCTAATCGCTCAACGAACGGATAGATAGTGTTTATTGCCTAGCAACTCGATGATAGATAGCAGACCAATGCACCTCAATTAGCGATAT-3’; containing 41.1% of GC). Serial dilutions from 109 to 101 copies/mL were used to generate the calibration curves for the RT-qPCR assays. All samples were tested in triplicate.

When the patients refereed that they were vaccinated against YF in the last 28 days before starting of symptoms and a putative YF vaccinal viscerotropical was suspected with plausible incubation period, RT-qPCR with primers and probes specific for vaccinal strains was carried out.^19^

The reactions were performed in 96-well plates and run on ABI Prism 7500 Fast real-time RT-qPCR system (Applied Biosystems, Thermo Fisher Scientific Brand, Carlsbad, CA, USA) under the following thermal cycling program: 50ºC for 30 min, 95ºC for 2 min, and then 45 cycles of 95ºC for 15s, and 55ºC for 1 min.

*Partial sequencing of YFV envelope gene* - The primers used to amplify YFV envelope (E) gene were previously described by Nunes et al.^20^ RT and first round PCR were performed using the SuperScript ® III One- Step RT-qPCR System with Platinum Taq High Fidelity kit (Invitrogen, Thermo Fisher Brand, Carlsbad, USA), and the primers YFV 975F and YFV 1312R. Second round hemi-nested RT-qPCR was carried out with Platinum Taq DNA polymerase (Invitrogen, Thermo Fisher Brand, Carlsbad, USA), using the same primers combination.

Positive samples were sequenced by Sanger method to determine the viral genotype. The 317bp RT-qPCR products were directly sequenced with an ABI PRISM terminator cycle sequencing kit v3.1 on an ABI 3500 DNA sequencer (Applied Biosystems, Thermo Fisher Scientific Brand, Carlsbad, CA).

*Phylogenetic analysis* - The sequence obtained was aligned with published reference sequences from the GenBank using the software BioEdit (v. 7.0.8) and the integrated CLUSTAL W program.^21^ A phylogenetic tree was constructed using Maximum Likelihood method using general time reversible (GTR) as substitution model and 500 bootstrap replications, using sequences from South America and Africa as references from the different genotypes.^22^

*Statistical analysis* - In order to determine which variables were associated with death, patients were classified in two groups: “survival” or “death” during hospitalisation. Continuous variables were tested by t Test (difference between means for two independent groups), and categorical variables were tested for association with death by Chi-square Test. After performing these univariate tests, variables presenting p < 0.20 were selected to enter a binomial logistic regression, backward stepwise method, in order to determine which variables best predict death combined in the logistic model, using the Wald Test to calculate the p-value for each variable coefficient entered in the model for each regression step. Odds ratio (OR) and 95% confidence interval (CI) were estimated for each variable included in the logistic model. Hosmer and Lemeshow test was applied to verify if observed and expected number of deaths were significantly different in each step of the logistic regression process. Nagelkerke coefficient of determination (R^2^) was calculated for each step of the logistic regression process in order to evaluate the degree of death explanation by the combination of variables in each step of logistic regression. Receiver operating characteristic (ROC) curves and the respective areas under the curve (AUC) were calculated for variables which remained statistically significant in predicting death in the last step of the logistic regression process. Survival curves were obtained by Kaplan-Meyer (K-M) test using the same independent variables selected from the logistic regression in its last step. Breslow test was used for comparison between the curves. All statistical analyses were carried out using SPSS version 21.0.

## RESULTS

*Laboratorial assays* - Among the 94 cases with YF patients that were admitted to the ICU, 62 were selected for the current analysis, because for 32 patients complete laboratorial data were not available. The results of these assays are shown in the Table I.

*Detection and quantification of YFV-RNA in serum and urine samples* - YFV-RNA was detected in all patients. In fifty-nine, it was detected in serum samples, but only three of them, YFV-RNA was just detected in urine samples. Only one patient has been previously vaccinated against YF in the past 28 days, but he was not infected with the YF vaccinal strain.

Viral load median was 6.71 log10 copies/mL (range 3.20 to 9.93 log10 copies/mL) among patients who were hospitalised during the first five days of symptoms, and 5.77 log10 copies/mL (range 3.40 to 8.48 log10 copies/mL) among patients who have been hospitalised more than five days after the first symptoms - p = 0.0002, 95%CI. On the other hand, as shown in Table I, viral load medians were not different between patients who survived and those who died.

**TABLE I:** Demographic and clinical laboratory data for 62 patients with severe yellow fever (YF), classified according to their outcome (survival or death). Univariate analysis.

Parameter	Survival (n = 21)	Death (n = 41)	p value
Age (years), median (IQR)	42 (27.5 - 48)	45 (35.5 - 58)	0.208
Gender, n (%) Male	16 (32%)	34(68%)	0.525
Female	5 (41.7%)	7 (58.3%)	
Days of symptoms^*^ , median (IQR)	6 (4.5 - 7)	5 (4 - 7)	0.489
ALT (U/L), median (IQR)	2,694 (1,416 - 3,642)	5,009 (3,242 - 7,734)	< 0.0001
AST (U/L), median (IQR)	3,384 (2,333 - 5,097)	11,350 (6752 - 15,820)	< 0.0001
AST/ALT, median (IQR)	1.33 (1.16 - 1.7)	2.04 (1.67 - 2.77)	< 0.0001
Total bilirubin (mg/dL), median (IQR)	3.38 (1.18 - 5.58)	5.85 (4.16 - 8.09)	< 0.0001
CKD-EPI (mL min^-1^ 1.73 m^-2^), median (IQR)	85 (65.5 - 114)	11 (6 - 25)	< 0.0001
Ammonia (μmol/L), median (IQR)	53 (41.5 - 62.5)	90 (62.5 - 141.5)	< 0.0001
Lipase (U/L), median (IQR)	66 (49 - 139.5)	531 (159 - 1560)	< 0.0001
Factor V (%), median (IQR)	90 (62 - 121.5)	32 (11 - 42.5)	< 0.0001
INR, median (IQR)	1.33 (1.15 - 1.51)	2.5 (1.97 - 3.51)	< 0.0001
Lactate (mg/dL), median (IQR)	16 (11 - 21.5)	39 (24.5 - 61.75)	< 0.0001
Bicarbonate (mmol/L), median (IQR)	20.7 (18.95 - 23.4)	14.7 (11.7 - 18.7)	< 0.0001
Viral load (log10 copies/mL), median (IQR)	6.1 (5.47 - 7.05)	6.1 (5.53 - 7.22)	0.623

ALT: alanine aminotransferase; AST: aspartate transaminase; CKD-EPI: chronic kidney disease epidemiology collaboration; INR: international normalised ratio; IQR: interquartile range; p < 0.05 was considered significant. *: days of symptoms up to day of hospitalisation.

Patients who were discharged from the hospital continued to be followed-up in the outpatient clinic and are expected to be followed-up for at least 1 year. Seven patients had their urine and blood screened weekly for YFV until the test was negative. After the onset of symptoms, viraemia and viruria were present for a maximum period of 28 days and 47 days, respectively.

**Fig. 1:**
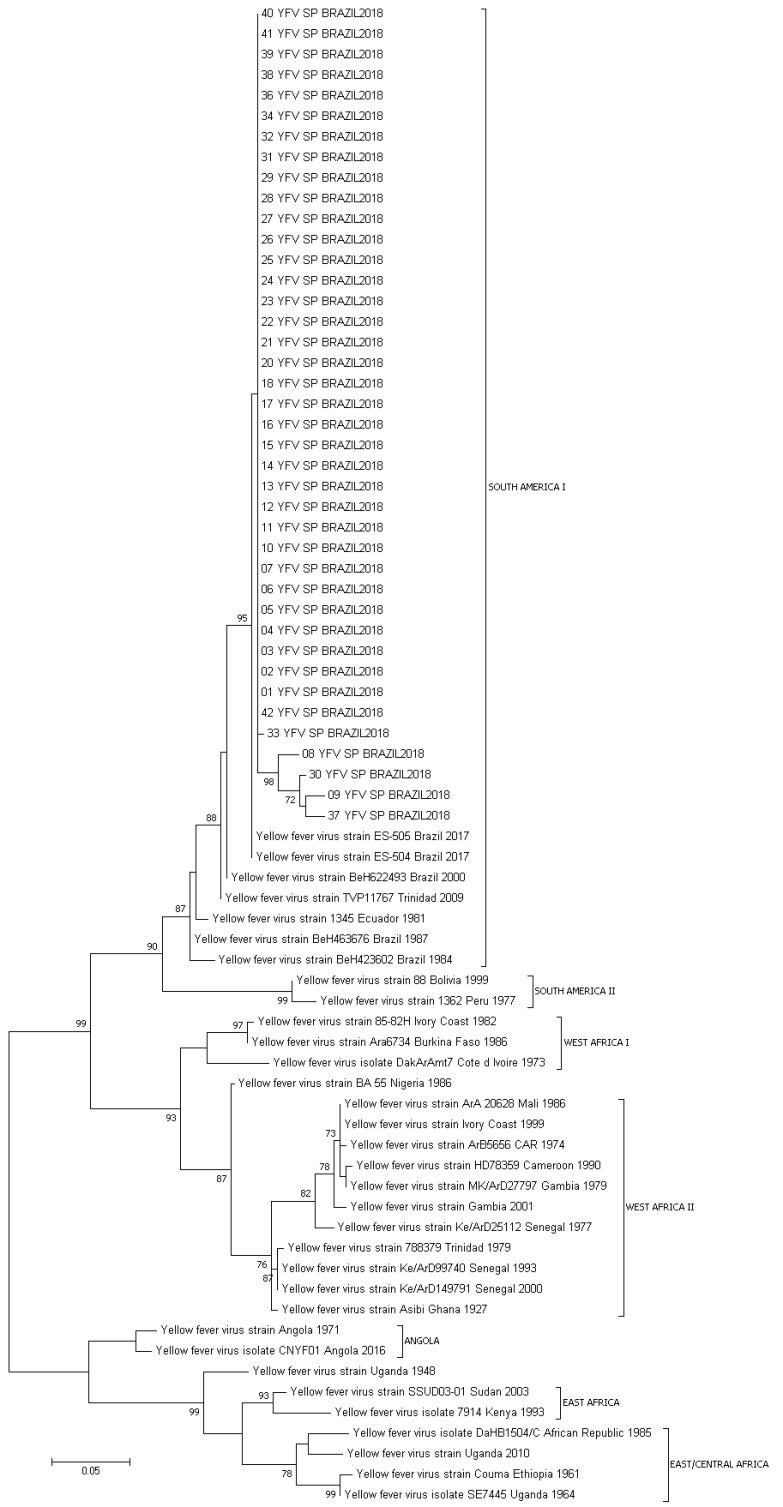
yellow fever virus (YFV) genotyping based on phylogenetic analysis using Maximum Likelihood method using general time reversible (GTR) as substitution model and 500 bootstrap replications.

*Phylogenetic analysis of YFV envelope gene and genotyping* - All isolated strains were characterised as YFV South American I (subclade 1E). São Paulo samples clustered together with the strains found in 2017 in the Brazilian states of Minas Gerais and Espírito Santo (Fig. 1).^23^

*Univariate analysis* - After running univariate analysis comparing patients who survived hospitalisation and those who died, 12 explanatory variables were selected for logistic regression (p < 0.20) as predictors of death, namely: age, CKD-EPI, ammonia, lipase, factor V, total bilirubin, INR, AST, ALT, AST/ALT ratio, lactate and bicarbonate (Table I).

*Logistic regression* - Binomial logistic regression was performed using death as the dependent variable and the 12 independent variables listed above. After 11 steps, only two independent variables remained statistically significant in the logistic regression model: lipase (OR = 1.018; 95%CI = 1.007-1.030; p = 0.002) and factor V (OR = 0.955; 95%CI = 0.929-0.982; p = 0.001). By the model, lipase combined with factor V explained 77.8% (Nagelkerke *R2*) of death prediction from YF and correctly classified 88.5% of death/survival cases. The AST/ALT ratio was excluded in the 10th step of the regression backward process (OR = 3.790; 95%CI = 0.958-14.996; p = 0.058). The complete results of the logistic regression are shown in Table II.

**TABLE II:** Complete results of the binomial logistic regression, backward stepwise methods, showing the statistical tests which evaluate the significance of variables coefficients and the model quality for each step of the process

Step	Model variables	Variable coefficient	Wald test for coefficients	Odds ratio	95%CI	Nagelkerke R^2^	Hosmer & Lemeshow	Correct prediction (%)
			*z*				*p*	χ^2^	p	death	survival	global
1	Age	0.108	1.962	0.161	1.113	0.958 - 1.294	0.914	20.677	0.004	97.5	95.2	96.7
	CKD	0.050	1.207	0.272	1.051	0.962 - 1.149						
	Ammonia	0.092	1.448	0.229	1.096	0.944 - 1.274						
	Lipase	0.041	4.388	0.036	1.042	1.003 - 1.082						
	Factor V	- 0.149	3.007	0.083	0.862	0.728 - 1.020						
	Bilirubin	- 0.295	0.167	0.683	0.744	0.181 - 3.067						
	INR	2.608	1.478	0.224	13.573	0.203 - 909.12						
	ALT	- 0.001	0.467	0.494	0.999	0.997 - 1.001						
	AST	0.00002	0.002	0.964	1.000	0.999 - 1.001						
	AST/ALT	3.162	1.569	0.210	23.609	0.168 - 3324.3						
	Lactate	- 0.231	3.455	0.063	0.794	0.622 - 1.013						
	Bicarbonate	- 0.582	2.204	0.138	0.559	0.259 - 1.205						
2	Age	0.108	2.196	0.138	1.115	0.966 - 1.287	0.914	20.528	0.005	97.5	95.2	96.7
	CKD	0.051	1.543	0.214	1.052	0.971 - 1.140						
	Ammonia	0.093	1.527	0.217	1.097	0.947 - 1.271						
	Lipase	0.041	4.589	0.032	1.042	1.003 - 1.081						
	Factor V	- 0.149	3.073	0.080	0.861	0.729 - 1.018						
	Bilirubin	- 0.292	0.166	0.683	0.747	0.183 - 3.041						
	INR	2.646	1.788	0.181	14.100	0.292 - 681.94						
	AST	- 0.001	2.089	0.148	0.999	0.998 - 1.000						
	AST/ALT	3.095	2.351	0.125	22.088	0.422 - 1154.72						
	Lactate	- 0.231	3.509	0.061	0.794	0.623 - 1.011						
	Bicarbonate	- 0.584	2.298	0.130	0.558	0.262 - 1.187						
3	Age	0.104	2.251	0.134	1.110	0.969 - 1.271	0.913	21.527	0.003	97.5	95.2	96.7
	CKD	0.052	1.566	0.211	1.053	0.971 - 1.142						
	Ammonia	0.094	1.673	0.196	1.099	0.953 - 1.268						
	Lipase	0.043	4.816	0.028	1.044	1.005 - 1.084						
	Factor V	- 0.171	5.180	0.023	0.843	0.727 - 0.976						
	INR	2.371	1.648	0.199	10.708	0.287 - 399.97						
	AST	- 0.001	2.939	0.086	0.999	0.998 - 1.000						
	AST/ALT	3.398	2.919	0.088	29.916	0.606 - 1476.07						
	Lactate	- 0.252	4.401	0.036	0.777	0.614 - 0.984						
	Bicarbonate	- 0.541	2.316	0.128	0.582	0.290 - 1.169						
4	Age	0.048	0.928	0.335	1.050	0.951 - 1.158	0.899	1.416	0.994	97.5	90.5	95.1
	Ammonia	0.052	0.938	0.333	1.053	0.949 - 1.169						
	Lipase	0.028	4.778	0.029	1.028	1.003 - 1.054						
	Factor V	- 0.123	4.768	0.029	0.884	0.792 - 0.988						
	INR	2.150	1.214	0.271	8.584	0.187 - 393.1						
	AST	- 0.001	1.570	0.210	0.999	0.999 - 1.000						
	AST/ALT	1.973	1.722	0.189	7.191	0.378 - 136.91						
	Lactate	- 0.175	3.972	0.046	0.839	0.707 - 0.997						
	Bicarbonate	- 0.163	1.101	0.294	0.849	0.626 - 1.152						
5	Ammonia	0.056	1.241	0.265	1.058	0.958 - 1.168	0.891	0.602	1.000	95.0	90.5	93.4
	Lipase	0.026	5.207	0.022	1.026	1.004 - 1.049						
	Factor V	- 0.107	4.848	0.028	0.898	0.817 - 0.988						
	INR	1.722	0.887	0.346	5.596	0.155 - 201.715						
	AST	- 0.001	1.355	0.244	0.999	0.999 - 1.000						
	AST/ALT	2.645	3.844	0.050	14.087	1.001 - 198.292						
	Lactate	- 0.175	3.992	0.046	0.840	0.707 - 0.997						
	Bicarbonate	- 0.129	0.935	0.333	0.879	0.677 - 1.141						
6	Ammonia	0.057	1.764	0.184	1.059	0.973 - 1.151	0.878	2.544	0.960	95.0	90.5	93.4
	Lipase	0.022	4.749	0.029	1.022	1.002 - 1.043						
	Factor V	- 0.091	5.093	0.024	0.913	0.844 - 0.988						
	AST	0.000191	0.396	0.529	1.000	0.999 - 1.000						
	AST/ALT	2.819	4.860	0.027	16.768	1.367 - 205.637						
	Lactate	- 0.146	3.775	0.052	0.864	0.746 - 1.001						
	Bicarbonate	- 0.124	1.119	0.290	0.883	0.702 - 1.112						
7	Ammonia	0.043	1.506	0.220	1.043	0.975 - 1.117	0.875	2.597	0.957	97.5	90.5	95.1
	Lipase	0.019	5.791	0.016	1.019	1.004 - 1.035						
	Factor V	- 0.077	6.383	0.012	0.926	0.872 - 0.983						
	AST/ALT	2.675	4.415	0.036	14.516	1.197 - 176.047						
	Lactate	- 0.122	4.541	0.033	0.885	0.791 - 0.990						
	Bicarbonate	- 0.150	1.856	0.173	0.860	0.693 - 1.068						
8	Lipase	0.018	5.842	0.016	1.018	1.003 - 1.034	0.859	6.691	0.570	92.5	90.5	91.8
	Factor V	- 0.078	7.483	0.006	0.925	0.875 - 0.978						
	AST/ALT	2.798	4.519	0.034	16.404	1.244 - 216.353						
	Lactate	- 0.080	2.530	0.112	0.923	0.837 - 1 019						
	Bicarbonate	- 0.064	0.638	0.424	0.938	0.802 - 1.097						
9	Lipase	0.019	6.320	0.012	1.019	1.004 - 1.034	0.854	6.840	0.554	92.5	85.7	90.2
	Factor V	- 0.081	8.888	0.003	0.923	0.875 - 0.973						
	AST/ALT	2.254	5.360	0.021	9.523	1.413 - 64.178						
	Lactate	- 0.084	3.377	0.066	0.920	0.841 - 1.006						
10	Lipase	0.013	5.964	0.015	1.013	1.003 - 1.023	0.837	2.889	0.941	92.5	85.7	90.2
	Factor V	- 0.066	8.576	0.003	0.936	0.895 - 0.978						
	AST/ALT	1.332	3.605	0.058	3.790	0.958 - 14.996						
11	Lipase	0.018	9.456	0.002	1.018	1.007 - 1.030	0.778	2.234	0.973	87.5	90.5	88.5
	Factor V	- 0.046	10.243	0.001	0.955	0.929 - 0.982						

ALT: alanine aminotransferase; AST: aspartate transaminase; CI: confidence interval; CKD-EPI: chronic kidney disease epidemiology collaboration; INR: international normalised ratio.

**Fig. 2:**
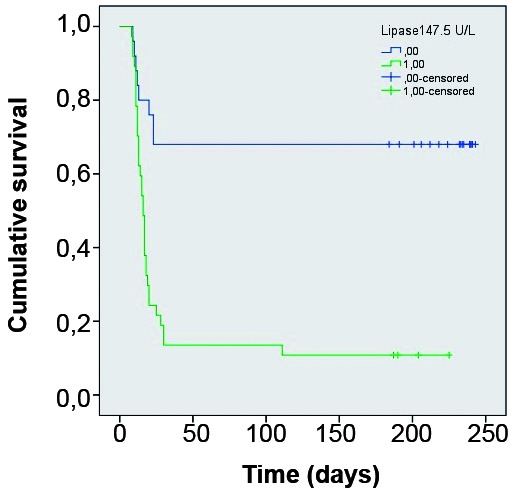
Kaplan Meyer survival curves comparing groups divided according Lipase below and above 147.5 U/L.

**Fig. 3:**
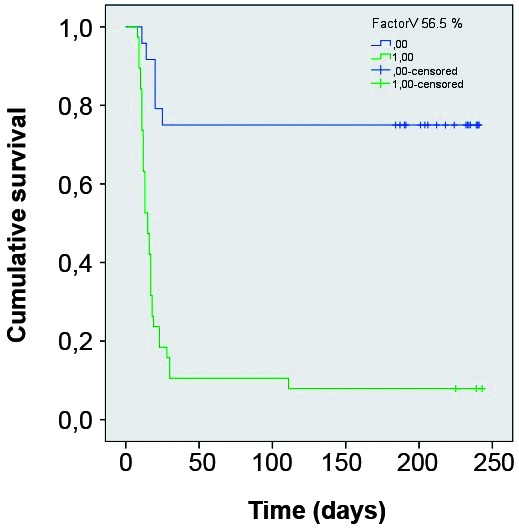
Kaplan Meyer survival curves comparing groups divided according Factor V below and above 56.5%.

The estimated lipase cut-off value that maximised sensitivity and specificity for death prediction was 147.5 U/L (AUC = 0.879). The estimated factor V cut-off value that maximised sensitivity and specificity for death prediction was 56.5% (AUC = 0.913). Using these cut-off values for lipase and factor V, K-M curves were constructed for these variables. In both cases the comparison between the curves using the Breslow test showed p < 0.001. (Figs 2-3).

## DISCUSSION

During the YF outbreak, we faced a disease that was very different from what we found described in the literature.^2^ ^,^ ^4^ ^,^ ^5^ ^,^ ^6^ Patients evolved very quickly and unexpectedly to death despite every effort to save them. Mortality was very high, even considering that these patients were critically ill.

Conditions such as pancreatitis, neurological events, and haemorrhagic phenomena in spite of normal coagulation tests behaved differently from what had been classically described in the literature.^4^ ^,^ ^24^ Pancreatitis in flavivirus has been described most frequently in cases of Dengue,^25^ ^,^ ^26^ West Nile^27^ ^,^ ^28^ and Rocio^29^ virus infections. A fatal YF case with pancreatic involvement was recently described in a Chinese patient who had acquired this infection in Angola,^30^ but pancreatic involvement due to YFV was not previously observed in rodent studies.^31^ ^,^ ^32^

In order to identify patients with a chance of developing severe disease, we tried to correlate clinical and laboratory variables with death. Clearly, the clinical outcome of the disease in critically ill patients is multifactorial.

Viral load was higher in patients who were hospitalised earlier when compared to those that have been hospitalised after the 5th day after the first symptoms. This might reflect the natural course of the disease, i.e., in the first days, viral load decreases as the disease progresses, due to the immune reaction against the virus. Nevertheless, the fact that the mean viral loads were not correlated with the outcomes (survival vs. death) suggests that in spite of the efficient immune control of the virus, disease evolution was more related to the viral effects on the different infected organs and to the global systemic inflammatory reaction during the first infection days. It reinforces that there are many gaps in the knowledge of the infectious process and the immune response against to relevant *Flaviviridae* in human beings that also need to be assessed studying cell biology, virology, and immunology.^33^

The continuous detection of virus in urine samples from some patients up to 47 days after the disease onset suggests that the virus may persist in the kidney longer than previously believed. These results agree with recent case report showing persistent viral detection in the urine 21 days after resolution of an acute YF infection.^34^ The long-term maintenance of YFV in the urine was also seen in other reports^35^ ^,^ ^36^ suggesting a viral related pathogenic process for the renal damage in the YF. Detection of YFV in urine is possible in some infected patients up to 47 days. It is noteworthy that CKD-EPI is also a laboratorial parameter that was also statistically relevant related to prognosis in the univariate analysis.

The variables that were associated with death were serum level of lipase and factor V. In specialised literature, pancreatitis did not appear as a relevant event but rather a haemorrhagic phenomenon.^31^ The absence of reporting pancreatitis as relevant in YF may be a consequence of the greater precariousness of medical care in the previous outbreaks of the disease described. Nevertheless, the finding of lipase as an independent variable associated with death reinforces the importance of pancreatitis for the outcome of the disease. The cut-offs suggested (lipase higher than 147.5 U/L and factor V lower than 56.5%) seem valuable to identify the most severe cases during outbreaks when most medical services may be flooded with large numbers of cases.

Classically the period of intoxication, in which symptoms are severe and viraemia is absent, is preceded by a period of remission. In our experience, remission did not occur and viraemia remained present during the severe presentation of the disease. This changes the classic view of YF as a markedly biphasic disease with a short duration viremia.^4^ ^,^ ^5^ ^,^ ^6^

An increase of AST/ALT ratio variable was not confirmed as an independent variable (p = 0.058), but the statistical trend confirms what is seen clinically, i.e., this inversion is related to the generalised involvement of different organs that can also increase AST levels (spleen, heart, kidneys, intestines and pancreas) besides the important effects on the liver tissue.

A limitation of this study is the absence of data from patients who were not critically ill, however these findings point to a new and better understanding of this deadly and under-studied disease among the most severe patients.

In conclusion, YF acute severe cases show a generalised involvement of different organs (liver, spleen, heart, kidneys, intestines and pancreas), and different parameters were related to outcome. Factor V and lipase are independent variables associated with death, reinforcing the importance of haemorrhagic events due to fulminant liver failure and pointing to pancreatitis as a relevant event in the outcome of the disease.
